# CDC Grand Rounds: Improving Medication Adherence for Chronic Disease Management — Innovations and Opportunities

**DOI:** 10.15585/mmwr.mm6645a2

**Published:** 2017-11-17

**Authors:** Andrea B. Neiman, Todd Ruppar, Michael Ho, Larry Garber, Paul J. Weidle, Yuling Hong, Mary G. George, Phoebe G. Thorpe

**Affiliations:** ^1^Division for Heart Disease and Stroke Prevention, National Center for Chronic Disease Prevention and Health Promotion, CDC; ^2^Rush University, College of Nursing, Chicago, Illinois; ^3^Veteran’s Administration Medical Center, Denver, Colorado; ^4^University of Colorado Denver; ^5^Reliant Medical Group, Worcester, Massachusetts; ^6^U.S. Public Health Service Commissioned Corps, Division of HIV/AIDS Prevention, National Center for HIV/AIDS, Viral Hepatitis, STD, and TB Prevention, CDC; ^7^Office of the Associate Director for Science, CDC.

Adherence to prescribed medications is associated with improved clinical outcomes for chronic disease management and reduced mortality from chronic conditions (*1*). Conversely, nonadherence is associated with higher rates of hospital admissions, suboptimal health outcomes, increased morbidity and mortality, and increased health care costs (*2*). In the United States, 3.8 billion prescriptions are written annually (*3*). Approximately one in five new prescriptions are never filled, and among those filled, approximately 50% are taken incorrectly, particularly with regard to timing, dosage, frequency, and duration (*4*). Whereas rates of nonadherence across the United States have remained relatively stable, direct health care costs associated with nonadherence have grown to approximately $100–$300 billion of U.S. health care dollars spent annually (*5*,*6*). Improving medication adherence is a public health priority and could reduce the economic and health burdens of many diseases and chronic conditions (*7*).

## Understanding Medication Nonadherence

Medication adherence is a complex behavior influenced by factors along the continuum of care, relating to the patient, providers, and health systems (*8*). Patient-related factors include unintentional factors, which often worsen with increasingly complex medication regimens (e.g., forgetting to take medication or obtain refills, or inadequate understanding of dose or schedules); and intentional factors (e.g., active decision to stop or modify a treatment regimen based on ability to pay, beliefs and attitudes about their disease, medication side effects, and expectations for improvement) (*9*) (Figure). Additional patient-related barriers include lack of engagement in treatment decisions, impaired cognition (e.g., related to aging or disease), substance abuse, depression, and other mental health conditions. Provider-related factors include barriers to communicating with patients and their caregivers, complex dosing regimens, and limited coordination of care among multiple providers. Health care system and service delivery factors include limited access to an appropriate provider for prescriptions or refills, restricted drug coverage, high costs and copayments, unclear medication labeling and instructions, limited availability of culturally appropriate patient education materials, and inadequate provider time to review benefits, risks, and alternatives to prescribed medications.

**FIGURE:**
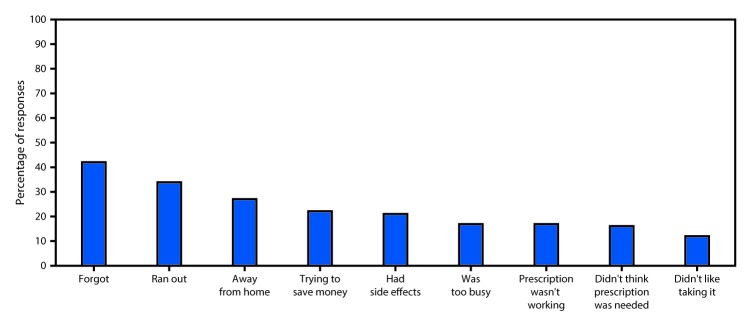
Self-reported reasons* for nonadherence to recommended medication regimens — United States, 2013 **Source:** Medication Adherence in America: A National Report 2013. Adapted with permission. https://www.ncpanet.org/pdf/reportcard/AdherenceReportCard_Abridged.pdf. * Participants could provide more than one response, and as such, categories are not mutually exclusive.

## Innovative Strategies to Improve Medication Adherence for Chronic Disease Management

Successful efforts to improve rates of adherence often incorporate multiple strategies across the continuum of care. A proven cost-effective strategy to reducing unintentional nonadherence is the use of pillboxes and blister packs to organize medication regimens in clear and simple ways (*10*). Combining the ease of packaging with effective behavioral prompts, such as electronic pill monitors that can remind patients to take their medication and provide messages to health care providers when a scheduled drug-dose is missed, supports increased medication adherence (*11*).

Interventions that include team-based or coordinated care have been shown to increase adherence rates. In a recent study, patients assigned to team-based care, including pharmacist-led medication reconciliation and tailoring; pharmacist-led patient education; collaborative care between pharmacist and primary care provider or cardiologist; and two types of voice messaging (educational and medication refill reminder calls) were significantly more adherent with their medication regimen 12 months after hospital discharge (89%) compared with patients not receiving team-based care (74%). Patients reported that team-based care improved their comfort in asking clarifying questions, raising concerns about their medication regimen, and collaborating in developing their treatment plan (*12*,*13*).

Lowering economic barriers to prescribed medications also improves adherence rates. In 2007, Pitney-Bowes Corporation employees and beneficiaries with diabetes or vascular disease increased their medication adherence rates increased by 3%–4% after the company eliminated or reduced health plan copays for cholesterol-lowering statins and the antiplatelet medication, clopidogrel (used to prevent heart attacks and strokes), compared with beneficiaries insured by another health plan with the same third-party prescription drugs administrator that did not reduce or eliminate copays for the same medications. These improvements, while modest, could result in significant cost savings in the prevention of acute events (e.g., hospitalizations) and progression of major chronic conditions if scaled to larger populations (*14*).

System-based strategies that address health disparities can improve clinical goals or reduce disease burden. For example, medication adherence is crucial for persons infected with human immunodeficiency virus (HIV), because treatment lowers the amount of virus circulating in the blood, which improves the patient’s health and reduces the risk of transmitting HIV to others by >90% (*15*). Interventions, such as CDC’s *Data to Care* (*16*) strategy, that identify and re-engage nonadherent patients in care by linking them through the health department, their care providers, or both, improve the health of the individual and achieve the public health benefit of reducing HIV transmission (*17*).

Advances in health information technology can also improve medication adherence. In a 2011 study, providers using electronic prescribing (e-prescribing) increased first-fill medication adherence by 10% compared with those using paper prescriptions (*18*). Some e-prescribing software can monitor prescriptions dispensed or unfilled in near real-time, as well as send patients prompts when a new or refill prescription is available. These data allow providers to review current medication use with patients during office visits, identify gaps or barriers to adherence, and discuss workable solutions.

Health information technology can also be used to show real-time impact of medication use on chronic conditions. Reliant Medical Group, a multispecialty group practice in Massachusetts, provided home blood pressure monitors to 200 of its patients. Patients uploaded blood pressure readings into their electronic health record. At office visits, providers were able to display trends of patients’ blood pressure, discuss barriers if blood pressure was not controlled and patients were not adherent, or add alternative drugs or lifestyle changes if pharmacy data indicated patients were adherent but their blood pressure was still poorly controlled. In addition, health information technology systems enabled providers to view medication coverage by insurer and choose lower cost medications. Reliant also made complex prescribing algorithms easier to follow by establishing and incorporating treatment protocols for hypertension into the electronic health record. Using these and other strategies (Box), Reliant improved its hypertension control rate from 68% in 2011 to 79% in 2014 and was recognized as a Million Hearts Hypertension Control Champion in 2015 (*19*).

BOX **Strategies used by Reliant Medical Group (Massachusetts) to improve adherence to blood pressure medication and increase hypertension control rates**

**Ensure that patient understands the benefits**

- Educate about harms of uncontrolled hypertension and benefits of controlling hypertension
- Make culturally appropriate education materials available
- Automatically print educational information in the After-Visit Summary if patient has diagnosis of hypertension
- Show patients graphs of their blood pressure trends during office visits and online electronic health record (EHR) portal. Use graphs to demonstrate challenges and successes with treatment regimens

**Choose lower cost medications**

- Use step-therapy protocols that are developed by a multidisciplinary team and are standardized across the organization
- Control access to pharmaceutical marketing
- Make the patient’s payer-specific formulary available in the EHR to inform medication selection
- Use generic medication substitution
- Provide assistance in paying for medications (e.g., RxAssist.org)
- Consult social workers to assist with adherence barriers

**Minimize medication complexity**

- Choose once-a-day and combination medications
- Engage in dialogue about costs versus convenience (e.g., pill-splitting can reduce cost but increase inconvenience)

**Monitor side effects**

- Be creative in addressing concerns
  - e.g., if concerned about swollen feet, use a diuretic, if appropriate
  - e.g., if concerned about medication causing abnormal potassium level, use a combined angiotensin-converting enzyme (ACE) inhibitor and a diuretic to normalize potassium
- When to monitor side effects
  - At visits
  - At prescription renewals, using a standard documentation template
  - After hospital discharge: automated alerts for new medications
- Consult pharmacists
  - For complex medication regimens or side effects
  - After hospital discharge regarding patients who are on high-risk medications

**Show effectiveness of the medications in lowering blood pressure**

- Empower patient to record blood pressure readings at home
- Provide booklets to record readings
- For patients with financial hardships, provide free home blood pressure monitors
- Offer free blood pressure clinics
- Automatically upload blood pressure readings into the EHR

**Monitor medication adherence**

- Encourage patients to document their medication-taking behavior
- Use EHR systems that can show medication fill history
- Automate adherence monitoring using payer medication claims
- Review adherence information during visits. Patients’ knowing that a clinician is monitoring adherence is at least as important as a patient seeing the results

## Opportunities in Medication Adherence Outcomes

Although a range of interventions have demonstrated improved medication adherence and health outcomes during the study period, few studies have shown that these benefits were maintained over time (*20*). Interventions that can sustain patient medication adherence are needed. One priority for developing sustainable strategies to improve medication adherence includes standardizing research methodology for both clinic and research settings. Currently, studies use a variety of measurement methods. Varying study methodologies prevents comparability across interventions, hinders wide application into clinical practice, and limits efforts that focus on patients with the greatest burden and need. Standardization might also help to understand both the dose-response and effectiveness of interventions over a longer time, increasing sustainability and reducing a waning effect at follow-up time points (*21*).

In addition, patient-specific tailored approaches to identifying reasons for nonadherence and aligning intervention efforts to address identified needs are needed. Outcomes might also be improved by recognizing populations at increased risk for nonadherence and addressing the broader reasons for their nonadherence, such as low health literacy. Health literacy is lower among the elderly, racial and ethnic minorities, and persons living in poverty (*22*). Interventions to improve medication adherence could be more effective if patient’s health literacy, cultural background, and language preference and proficiency are taken into account when designing communication and patient education materials.

## Conclusion and Comments

Medication adherence is critical to improving chronic disease outcomes and reducing health care costs. Successful strategies to improve medication adherence include 1) ensuring access to providers across the continuum of care and implementing team-based care; 2) educating and empowering patients to understand the treatment regimen and its benefits; 3) reducing barriers to obtaining medication, including cost reduction and efforts to retain or re-engage patients in care; and 4) use of health information technology tools to improve decision-making and communication during and after office visits. Understanding root causes of medication nonadherence and cost-effective approaches that are applicable in diverse patient populations is essential to increasing adherence and improving long-term health impact.
